# Natural Blends of Ethyl Cellulose Oleogels for Tunable Bioplastic Design

**DOI:** 10.1021/acsomega.5c11908

**Published:** 2026-01-27

**Authors:** Luca Cafuero, Marco Friuli, Muhammad Waheed, Christian Demitri, Alessandro Sannino, Carola Esposito Corcione, Leonardo Lamanna

**Affiliations:** † Department of Engineering for Innovation, 18976 Universita del Salento, Via Monteroni, Lecce, Apulia IT 73100, Italy; ‡ Department of Experimental Medicine, Universita del Salento, Via Monteroni, Lecce, Apulia IT 73100, Italy

## Abstract

Recently, ethyl cellulose Oleogels have been proposed as gel state, biobased, and biodegradable thermoplastics. This study explores new Oleogel formulations by tailoring the oil phase to improve material customizability. Four classes of biobased oily molecules were selected: a hydroxylated triglyceride—castor oil, a free fatty acid—oleic acid, a phenolic lipid—cardanol, and a citric acid derivative—tributyl citrate. These formulations allowed us to assess the influence of each molecule on the chemical, physical, and mechanical properties of the Oleogels. Cardanol provided the most effective plasticization, increasing elongation at break by 5.6-fold, while oleic acid induced the largest reduction in glass-transition temperature (Δ*T*_g_ ∼ 50 °C). Castor oil, in contrast, achieved a balance between elasticity and stability, preserving the tensile strength and thermal resistance. The study presents a comprehensive rheological, mechanical, and physicochemical characterization of these thermoplastic Oleogels, highlighting their potential as a new class of tunable bioplastics.

## Graphical abstract

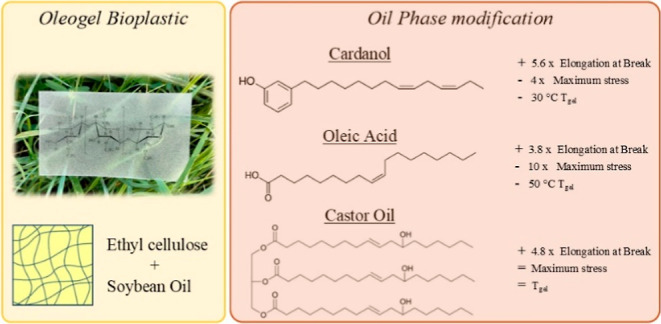

## Introduction

Oleogels (OGs) represent an interesting class of biomaterials that remain relatively unexplored in material science compared to hydrogels. In general, they consist of two main components: an oil phase, which acts as the matrix, and an oleogelator (or oil-structuring agent) that induces the formation of a three-dimensional network.^1,2^ Unlike the extensively studied hydrogels, whose tunability mainly relies on modifications of the polymer network or the aqueous environment (pH or ionic strength), Oleogels present distinct advantages. Their liquid phase is nonvolatile, and the oil-based continuous phase can be readily tailored by selecting or blending different oils. Currently, OGs are already used in several industrial sectors, particularly in the food industry for texturing and as substitutes for saturated fatty acids,^1^ and in pharmaceutics as excipients or carriers for controlled drug delivery systems.^1,3–7^

In 2024, ethyl cellulose (EC)-based Oleogels have been proposed by our group as a biobased and biodegradable Bioplastic, named *OleoPlast*, in a study published in Chemical Engineering Journal.^8^ *OleoPlast* proved to be processable using all major thermoplastic techniques, including injection molding, compression molding, extrusion, CNC milling, fused filament fabrication (3D printing), and micropatterning, thus paving the way for a new class of bioplastics suitable for diverse fields of use. The material exhibited properties that overcome one of the main limitations of bioplastics (e.g., PHA, PLA), whose processing temperatures are critically close to their degradation points.^9–11^ In Oleogels, the processing temperature (∼165 °C) is far below the degradation temperature (>350 °C), providing a much wider and safer thermal window. This feature enabled excellent recyclability, with mechanical properties largely preserved even after five recycling cycles, showing only a 5% reduction in maximum sustained stress.^8^ Moreover, *OleoPlast* proved biodegradable in seawater, as demonstrated by Biochemical Oxygen Demand (BOD) tests, with results comparable to PHB. The resulting materials exhibited a Young’s modulus ranging from 3 to 600 MPa, comparable to polyethylene (PE) and lower than that of bioplastics such as PLA and PHB, which are typically stiffer. However, the material demonstrated an elongation at break of around 20%, placing it between the cited bioplastics (∼3%) and the highly plastic PE (∼300%).^8^ *OleoPlast* is now being evaluated for a range of applications previously inaccessible to Oleogels, including packaging, where its permeability is in line with common bioplastics (OTR ∼ 5000 mL m^–2^ day^–1^; WVTR ∼ 400 mL m^–2^ day^–1^),^12^ electronics, as a substrate for flexible and edible devices,^13^ and agriculture,^14^ as a dispenser for pest management aimed at replacing more environmentally impactful silicones. However, to make EC-based Oleogels suitable for uses such as wearable electronics and packaging, their flexibility and tunability still need to be improved.

The previous study focused on the role of EC content and the use of widely available, low-cost vegetable oils (VOs) such as soybean, sunflower, and peanut oil. It demonstrated that a polymer concentration of 50% combined with soybean oil (SBO) offered the best trade-off among mechanical properties, stability, and processability and was therefore set as the reference sample, hereafter referred to as CTRL. In this study, four novel Oleogel formulations were investigated by modifying the oil phase while keeping the polymer content constant, aiming to expand their tunability and potential applications while preserving their biobased nature. The formulations incorporated four distinct classes of oily biomolecules: castor oil (CO), oleic acid (OA), cardanol (CAR), and tributyl citrate (TBC). The purpose of using these molecules was to assess how selected oils can modify the chemical and physical properties of the material.

Castor oil (*M*_w_ ∼ 933 g mol^–1^) is a triglyceride characterized by an exceptionally high content (∼90%) of ricinoleic acid, a fatty acid containing double bonds and reactive −OH groups capable of forming hydrogen bonds.^15–17^ Oleic acid (*M*_w_ ∼ 282 g mol^–1^) is a free fatty acid commonly found in most VOs; it consists of an 18-carbon monounsaturated chain and is widely used in the synthesis of biobased plasticizers.^18,19^ Cardanol (*M*_w_ ∼ 298 g mol^–1^) is an oil derived from cashew nutshell liquid (CNSL), featuring a phenolic ring with a long unsaturated aliphatic chain; it is already employed as a plasticizer for cellulose-derivative polymers.^20–23^ Tributyl citrate (TBC) (*M*_w_ ∼ 360 g mol^–1^) is an ester of citric acid. Citrate esters are typically used in food packaging, and TBC is already applied as a bioplasticizer for different biomaterials.^24–27^ Two concentrations, 25.0 and 37.5%, were investigated to assess their effect on the mechanical, thermal, and physicochemical properties of the Oleogel (Table 1). All new formulations exhibited lower stiffness than the CTRL, with the cardanol-based one showing a more than 5.6-fold increase in elongation at break and a one-order-of-magnitude reduction in both Young’s modulus and tensile strength. Moreover, cardanol and oleic acid markedly reduced the gel temperature between 30 and 50 °C, allowing processing at lower temperatures. FTIR and contact angle analyses revealed no significant differences in chemical or surface behavior compared to CTRL.

**Table 1:** Coded Name of Different Oleogels Produced and Characterized

material Id	modifier	percentage
CTRL	-	-
CAR 25.0	cardanol	25.0
CAR 37.5	cardanol	37.5
CO 25.0	castor oil	25.0
CO 37.5	castor oil	37.5
OA 25.0	oleic acid	25.0
OA 37.5	oleic acid	37.5
TBC 25.0	tributyl citrate	25.0
TBC 37.5	tributyl citrate	37.5

## Results and Discussion

Young’s modulus, maximum tensile strength (σ_max_), and maximum elongation (ε_max_) were measured for all formulations, Figure 1. TBC was not considered in the analysis due to leaching, which was not observed with the other formulations. All results were compared to those of the control formulation containing soybean oil (CTRL).

**Figure 1:**
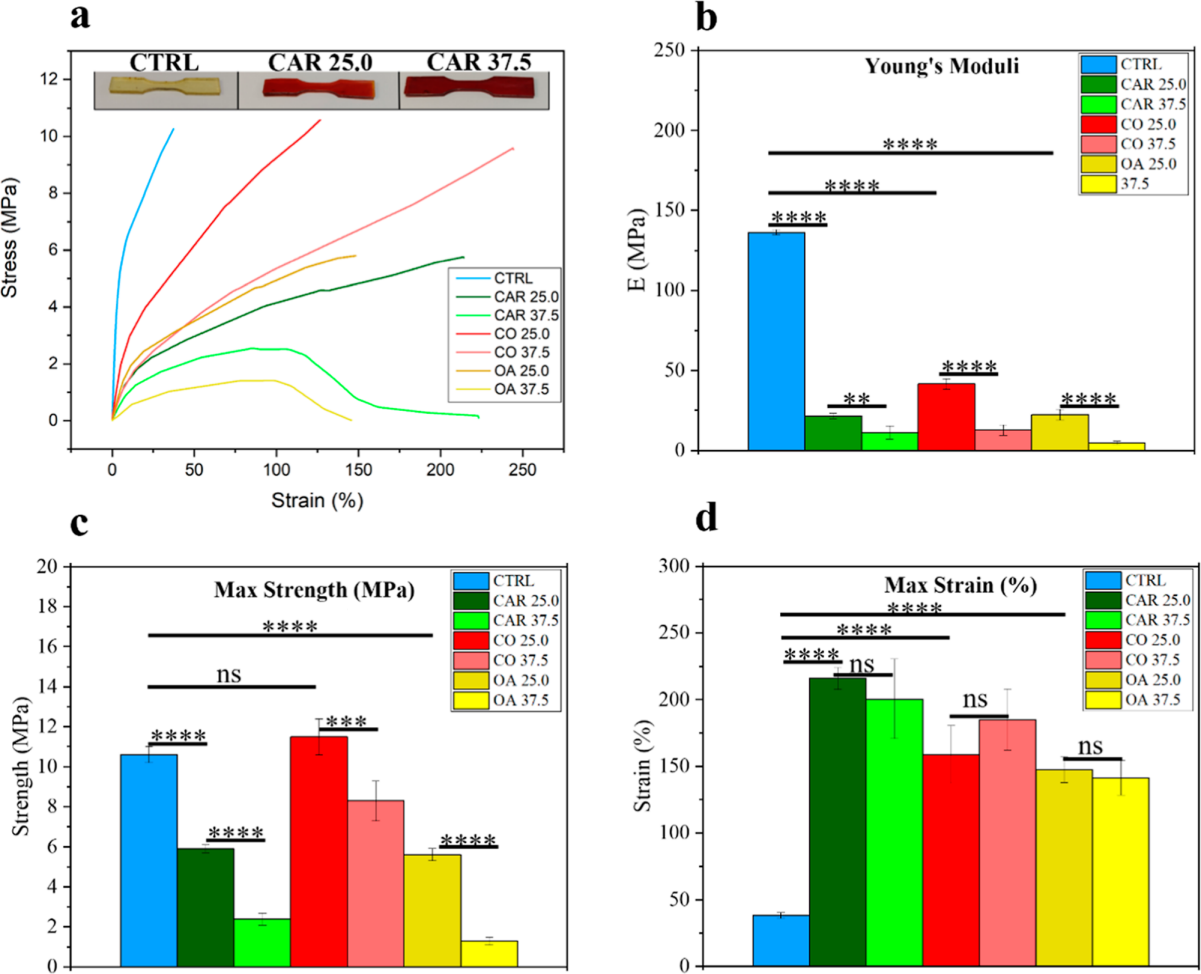
Mechanical characterization of the investigated blends: (a) representative stress–strain curves; (b) elastic modulus; (c) maximum stress; and (d) maximum strain.

The new formulations showed a lowering of Young’s Modulus by dropping it by 1 order of magnitude (from >130 MPa to ∼5–41 MPa) while the strain at break increased up to ∼5.6 times for cardanol. This trend aligns with classical plasticization theory, according to which stiffness and tensile strength decrease as ductility increases.^28^ OA and CAR significantly reduced σ_max_ with the OA 37.5 formulation, lowering it to ∼4.8 MPa. Both acted as classical plasticizers, with the most pronounced effects observed in strength and Young’s modulus (Figures 1a,b and S1), while their influence on elongation remained relatively minor. In contrast, CO exhibited a different behavior (Figures 1a,b and S1). Although it lowered the modulus (∼41 MPa), the material maintained a σ_max_ comparable to the CTRL. This behavior is likely due to the ricinoleic fatty acids in castor oil, whose −OH groups act as plasticizing agents by disrupting intrachain EC–EC hydrogen bonds and thereby increasing chain mobility. At the same time, these hydroxyl groups form new hydrogen bonds with EC (EC–Oil), which help preserve the overall network cohesion.^15^ As a result, the material becomes softer and more extensible while maintaining its tensile strength. TBC also acted as a plasticizer; however, its results were not considered for further characterization due to leaching.

The new *OleoPlast* blends were also characterized under cyclic compression at 25% strain over 10 cycles on a cylindrical sample (picture in Figure S2). The results confirmed the trends observed in the tensile tests: CTRL exhibited the highest stiffness, while all modified formulations showed a reduction in rigidity (Figure 2a). With each compression cycle, the material exhibited a reduction in stress at a given strain, as is typically observed in plastic materials. This effect was particularly pronounced during the first cycles, with all samples showing an approximate 10% loss in load-bearing capacity by the third cycle (Figure 4b). Among the tested formulations, OA showed the largest decrease in the maximum stress after cycling. Residual strain was also evaluated after each cycle as an indicator of the material’s ability to recover its original dimensions, with lower values corresponding to improved recovery performance (Figure 2a,b). CTRL showed the highest residual deformation, indicating greater structural obsolescence, with a significant difference compared to the other samples from the first cycle (Figure 2c). CO 37.5 formulation displayed the best cyclic recovery, with a residual strain of approximately 7% after 10 cycles, less than half that of CTRL. All other blends exhibited residual strains around 10% after 10 cycles. These behaviors are consistent with the expected plasticization mechanism, where the presence of low-molecular-weight molecules facilitates polymer chain mobility, leading to a reduction in accumulated stress and consequently to improved shape recovery. The case of castor oil is particularly significant, as it supports the hypothesis that CO not only acts as a plasticizer but also establishes weak hydrogen-bonding interactions with EC, generating a secondary oil–EC subnetwork that enhances both shape recovery and ultimate tensile strength.

**Figure 2:**
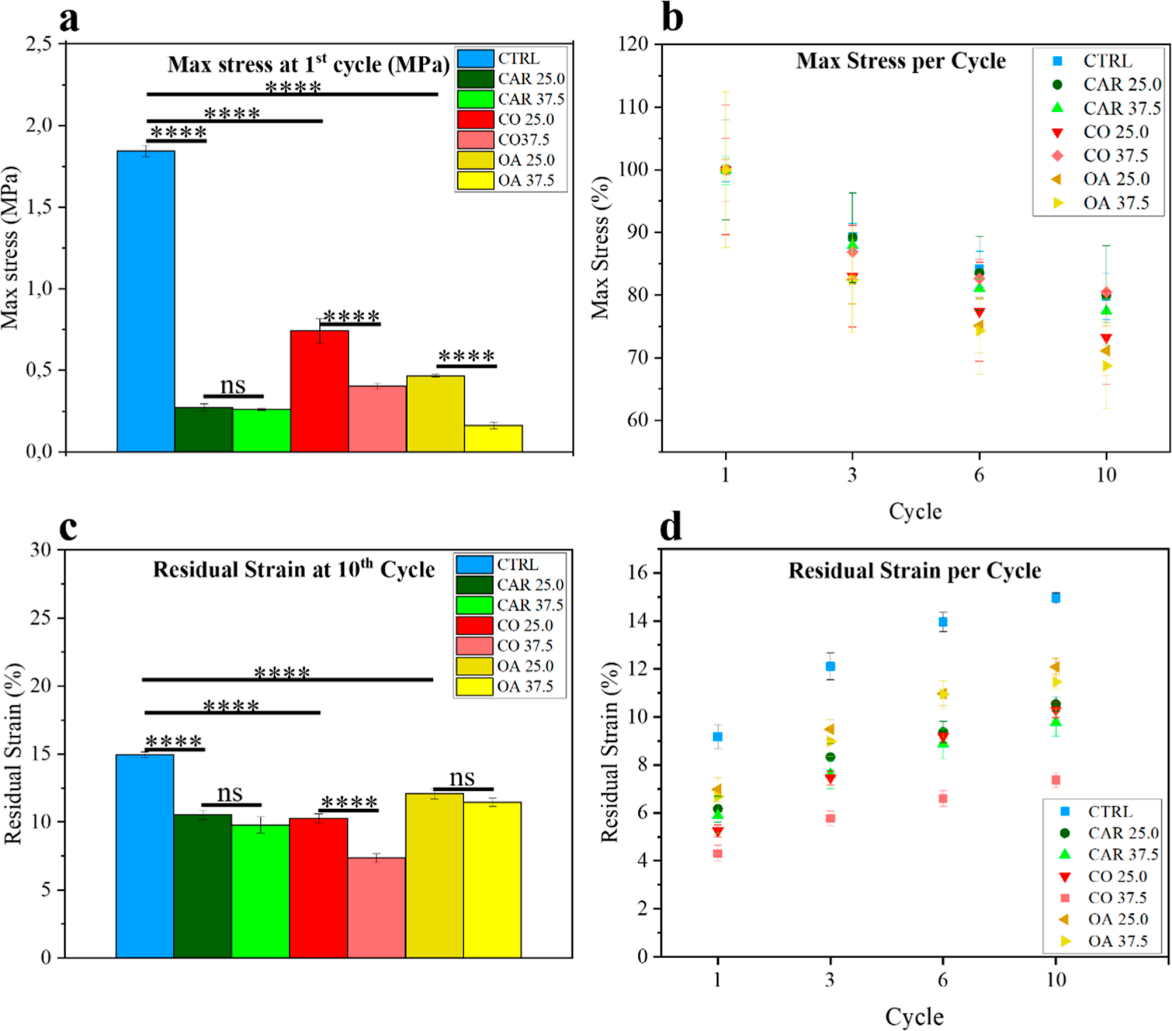
(a) Max stress in compression at first cycle for each formulation. (b) Progressive max stresses at selected cycles for all the blends normalized to the first cycle. (c) Residual strain after 10 cycles of compression. (d) Progressive residual strain at selected cycles.

**Figure 3:**
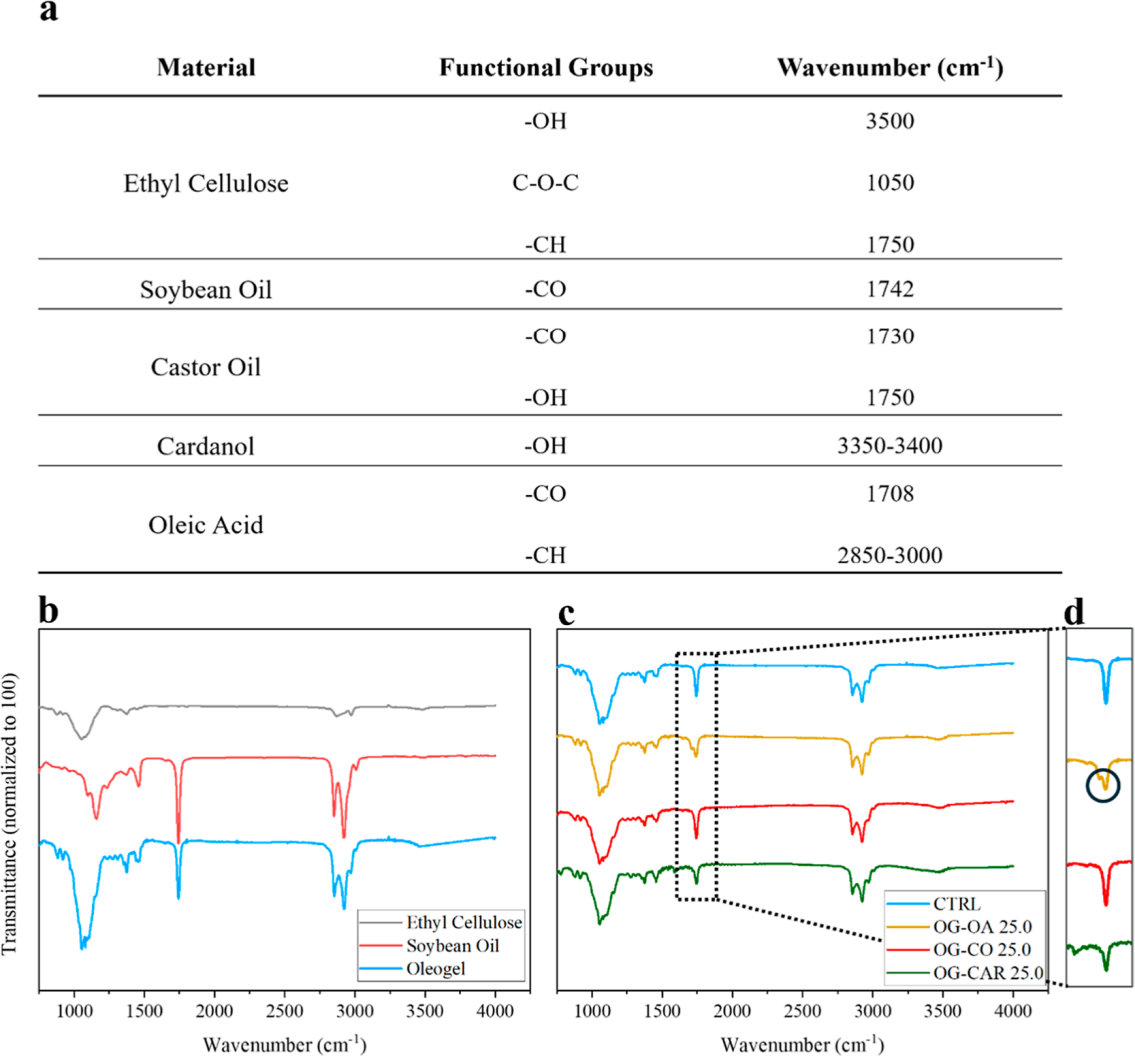
(a) All the characteristic groups of the materials used for different formulations are reported. (b) The FTIR spectra of EC, SBO, and the OG. (c) All the FTIR spectra for the different blends at 25% of concentration. (d) The double peak of the asymmetric stretch at 1708 cm^–1^ of –C=O of oleic acid and the peak of –C=O at 1742 of SBO.

**Figure 4:**
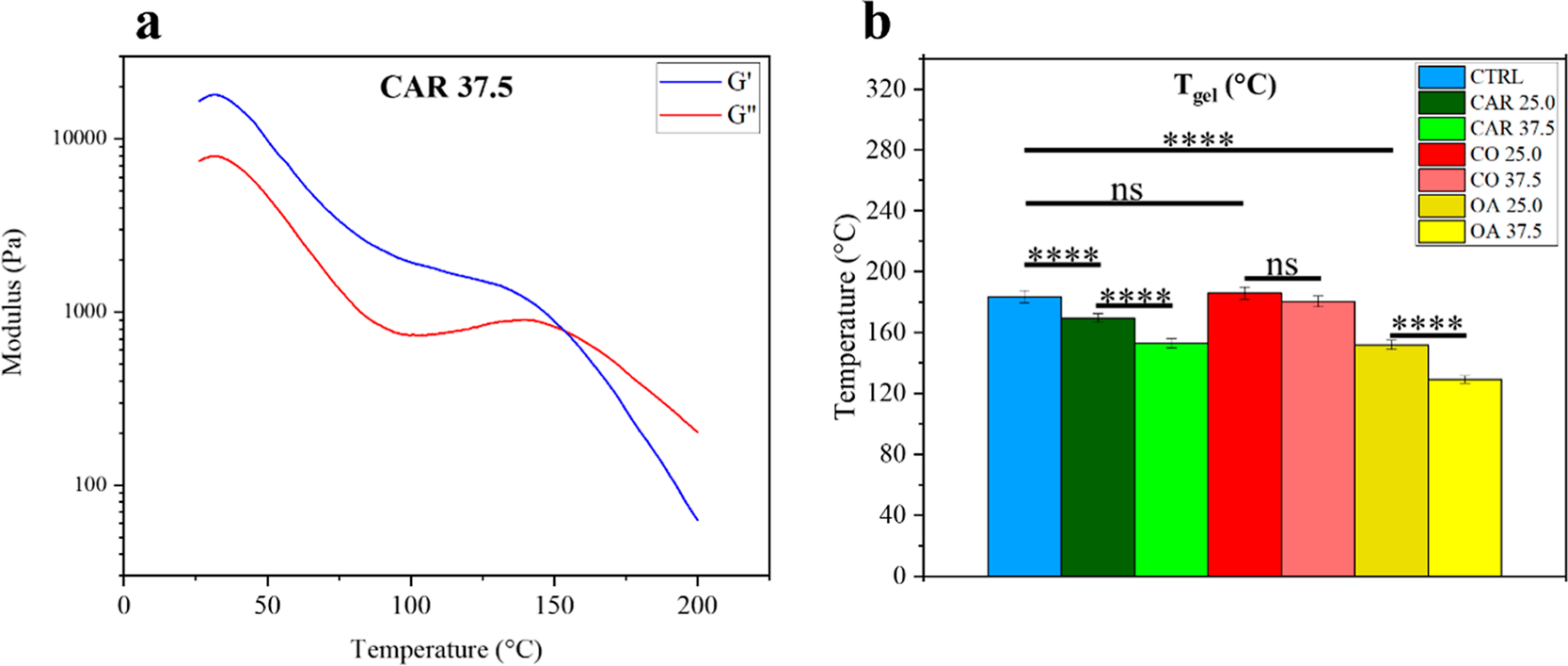
(a) The crossover point represents the temperature at which the material’s behavior becomes liquid-like. (b) In all cases but CO, the gelation temperature strongly lowered.

### Wettability Test

All new formulations showed no significant change in the water contact angle, which remained below 90° (Table 2 and Figure S3). Only oleic acid, at a higher concentration, produced a slight increase compared to the control, reaching 86°, an effect previously reported when OA was used as a plasticizer.^29,30^

**Table 2:** Contact Angle for Each Formulation

material	contact angle (°)
CTRL	69.0 ± 4
CAR 25.0	73.9 ± 2
CAR 37.5	68.0 ± 6
CO 25.0	59.0 ± 8
CO 37.5	73.6 ± 7
OA 25.0	65.5 ± 4
OA 37.5	86.5 ± 3

### FTIR-ATR

FTIR analysis was performed to determine whether the new formulations generated any chemical interactions between the oil phase and EC. By comparing the FTIR spectra of EC, SBO, and OG, it is evident that the Oleogel spectrum represents a superposition of the two components. The carbonyl stretching of SBO, visible at 1742 cm^–1^,^31^ and the O–H stretching band of EC at approximately 3500 cm^–1^,^32^ occur at the same wavenumbers in the OG spectra. A similar superposition is observed in all blended Oleogels (Figures S4 and S5). When comparing Oleogels prepared with the same biomolecule but at different ratios, no changes are detected other than variations in peak intensity (Figure S4). For instance, in the Oleogel containing oleic acid, the characteristic double peak arises from the overlap between the asymmetric C=O stretching of OA at 1708 cm^–1^ ^33^ and the corresponding C=O band of SBO at 1742 cm^–1^ (Figure 3d).^34^

### Dynamic Thermo-Mechanical Analysis (DMTA)

The gel temperature (*T*_gel_) is defined as the point at which a material passes from a solid-like to a liquid-like behavior and it is identified as the crossover point between the elastic modulus *G*′ and the viscous modulus *G*″.^35^ This parameter is fundamental to determining the appropriate processing temperatures of the material. The variation on *T*_gel_ is negligible in the blend with CO, while it varies when CAR and OA are present; further, the more they are present the more *T*_gel_ lowers (Figures 4a,b and S6). The EC Oleogel structure arises from two factors: (i) the ability of EC to form H-bonds, which stabilize the network and (ii) the affinity of EC ethoxy groups for apolar oil molecules.^36^ For instance, OA, being a smaller free fatty acid with respect to SBO, interferes with the polymer matrix by reducing the number of hydrogen bonds, thereby lowering the gelation temperature of the Oleogel. CAR shows a comparable but weaker effect; its rigidity from the aromatic ring and C=C bonds along the chain restricting flexibility.^37,38^ The transition between solid-like and liquid-like states is also confirmed by thermodynamic analysis through differential scanning calorimetry (DSC) (Figure S7), which reveals a second-order transition. However, this transition is not pronounced because of the gel state, as the material contains only 50 wt % polymer, an observation already demonstrated in previous work.^8^ CO shows a negligible effect on the *T*_gel_ of the Oleogel because its high ricinoleic acid content (∼90%) enables the formation of a large number of H-bonds, unlike OA and CAR, thus favoring the development of a stable structure,^39^ even between the oil and EC.

### Thermogravimetric Analysis (TGA)

The thermal stability of the new formulations was assessed by TGA, focusing on the temperatures corresponding to 5% (*T*_5_) and 50% (*T*_50_) weight loss, as well as on the residual mass at 500 °C (Figure 5a,b). Both CAR and OA significantly reduced the onset of degradation, with *T*_5_ decreasing by about 100 °C (∼220 °C for OA and ∼245 °C for CAR). The *T*_50_ values also decreased, reaching ∼350 °C for OA and ∼365 °C for CAR. In contrast, the CO formulation showed a degradation profile comparable to that of CTRL, consistent with its triglyceride structure, similar to that of SBO. The residual mass at 500 °C showed no substantial differences among the samples.

**Figure 5:**
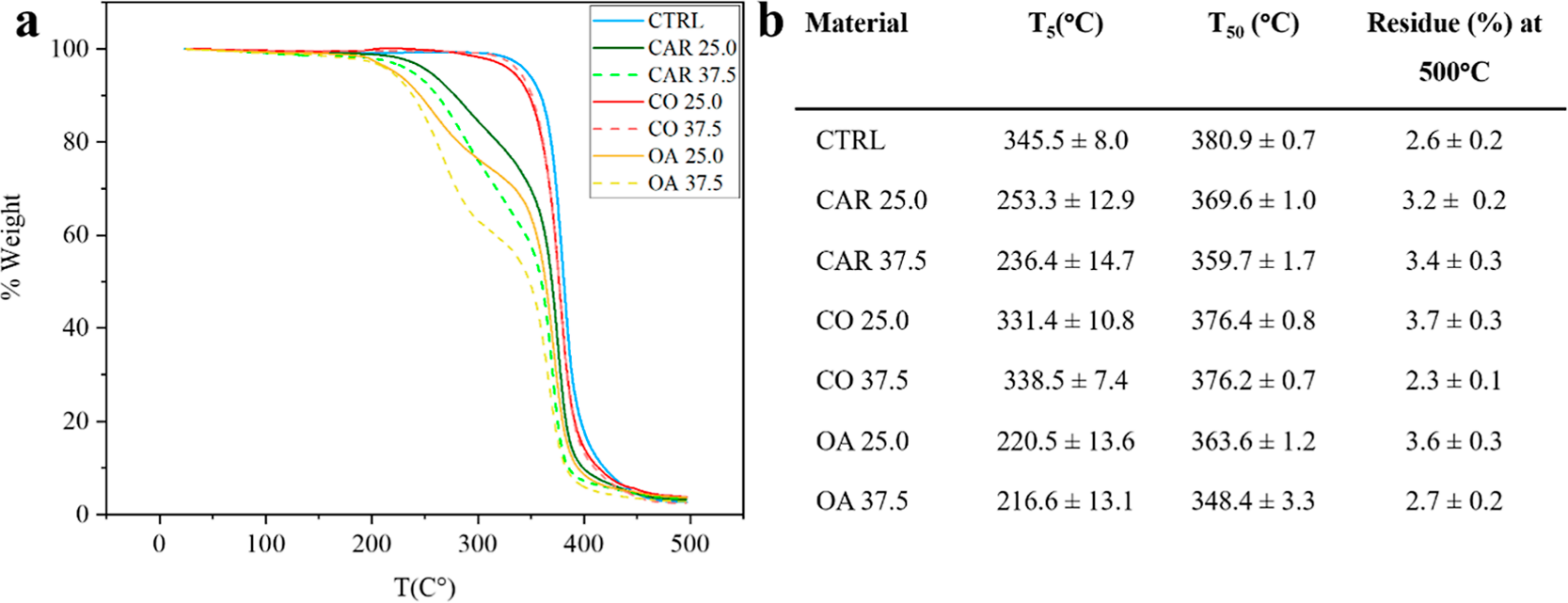
(a) TGA curves of all the materials underlines the different modes of degradation of the blends. (b) The initial (*T*_5_) and mid (*T*_50_) degradation temperatures and the final residue for all Oleogels’ blends are reported.

## Conclusions

This work demonstrated that the Oleogel, previously proposed by our group as a bioplastic named *OleoPlast*, based on EC and soybean oil, can be effectively tuned in its mechanical and physical properties simply by modifying the oil phase here through the exploration of different natural oleophilic molecules. Cardanol and oleic acid acted as plasticizers, increasing the elongation at break by approximately 5.6 times while reducing both the modulus and the sustained stress and lowering the gelation temperature by about 40 °C. Castor oil, on the other hand, produced interesting results, increasing the elongation at break by 4.8 times while maintaining both the maximum sustained stress and the glass-transition temperature. This behavior is attributed to its triglyceride structure, similar to soybean oil but enriched with hydroxyl groups that weaken EC–EC intrachain interactions while promoting EC–oil interactions, enabling higher chain mobility without loss of overall cohesion. In all cases, the formulations did not generate new chemical bonds but only weak interactions, as confirmed by FTIR analysis.

In the future, this bioplastic platform may be expanded by incorporating additional components, among others, phospholipids or mono- and diglycerides. Further studies will assess how these new formulations affect long-term aging and degradation as well as their potential use in packaging and electronics, where plasticity and customizability are key requirements.

## Methods

Ethyl cellulose was produced by ChemPoint (reseller of Dupont ETHOCEL Standard 100 Industrial) with viscosity in the range of 90–110 mPa·s and a degree of substitution of about 2.5 (48.9–49.5%). For the OG was employed commercially available soybean oil (SBO), specifically Desantis, composed by 9–13% of saturated fatty acid, 25–33% of monounsaturated fatty acid, and 55–65% of poly unsaturated fatty acid.^8^

Tributyl Citrate was produced by BCD Chemie under the name Citrofol B1, 99% pure. Oleic acid of technical grade 90% was purchased from Sigma-Aldrich. Commercial cold-pressed Castor Oil 100% pure was by Matt. Cardanol was purchased from Oltremare (Bologna, Italy) and was 99% pure.

The production of Oleogel followed the method we described in the previous work: vegetable oil and ethyl cellulose were mixed tighter in hot mixing until a homogeneous and viscous fluid was reached and then naturally cooled. The new blends of *OleoPlast* were produced by adding CO, OA, CAR, and TBC during the hot mixing phase.

To produce OG dogbone ASTM D638 Type V specimens, the material was grinded after being frozen using liquid nitrogen (to facilitate grinding avoiding overwarming). OG pellets were hot-pressed in a press type P7-91-PL at 165 °C and 25 bar (2.5 MPa) into a specifically shaped mold.

### Mechanical Characterization

The mechanical traction test was performed at room temperature on a Lloyd LR50K-plus machine on a dogbone ASTM D638 Type V. A speed of 5 mm/s at room temperature was used to execute the test, and it was repeated on 3 dogbone specimens until their failure.

### Cyclical Compression

The cyclical compression test was performed at room temperature on a Lloyd machine on cylindrical specimens in triplicate (4 mm in thickness and 14 mm in diameter) fabricated by hot-pressing with the same parameters as the dogbone specimen. The test consisted of 10 consecutive compression–decompression cycles. The compression stage was brought until 1 mm, which was 25%, at 0.5 mm/s of deformation starting from the original size, then the load was removed, then applied again without time gap between cycles. The tests were performed on a Lloyd LR50K-plus machine at room temperature.

### Dynamic Thermo-Mechanical Analysis (DTMA)

Gelation temperature was determined on a Malvern rheometer using a flat plate of 20 mm in diameter using a dynamic thermo-mechanical analysis (DTMA) with a temperature ramp 25–200 °C with a heating rate of 5 °C/min. The viscoelastic region was determined by an amplitude test, after which the strain was set at 0.04% at a frequency of 0.4 Hz. The test consisted of the monitoring the elastic and the viscous moduli (*G*′ and *G*″, respectively) by applying a monofrequency shear strain on cylindrical shaped samples (0.5 mm in thickness and 15 mm in diameter) obtained from a hot-pressing process previously described.

### Thermogravimetric Analysis (TGA)

A thermogravimetric analysis test was conducted on the TGA Q500 instrument in the range of 25–500 °C on pellet samples of 5–10 mg at a flux of 10 °C/min, on alumina crucibles. The tests were performed on an inert atmosphere with nitrogen flux of 50 mL/min on the OG pellets.

The thermal stability of the materials was evaluated based on three key points of the degradation curve. The first point, *T*_5_, corresponds to the temperature at which a 5% mass loss is recorded and is considered the onset of degradation. The second point, *T*_50_, represents the temperature at which 50% of the mass is lost.

### Differential Scanning Calorimetry/Differential Thermal Analysis (DSC/DTA)

The DSC–DTA tests were conducted on machine DSC Q2500 (TA Instruments), in a temperature range of −30 to 200 °C at a rate of 5 °C/min, on aluminum crucibles. The tests were performed under an inert atmosphere with nitrogen flux of 50 mL/min using some pellet specimens of 10–15 mg.

### Fourier Transform Infrared Spectroscopy Test (FTIR)

Fourier transform infrared spectroscopy (FTIR) analysis, performed with a Jasco 6300 FTIR spectrometer (JASCO Corporation, Tokyo, Japan), was used to characterize the single components and all of the Oleogels. Infrared spectra were recorded in the wavelength range between 750 and 3500 cm^–1^, 50 scans, and 4 cm^–1^ of resolution, by using ATR Pro One X with ZnSe crystals. The analyses were performed at room temperature.

### Wettability Test

The wettability test was performed using a sessile drop test at room temperature. The drop of distilled water (5 μL) was carefully laid on the surface of a thin film (1 mm thickness) obtained by a hot-pressing process. The image was captured by a camera, and the angles were measured by using First Ten Angstroms, FTA 1000 software (Newark, California, USA) equipped with a CDD camera. The test was repeated in triplicate.

### Statistical Analysis

The data were reported as mean ± standard deviation (SD) for the indicated number of experiments, each performed at least in triplicate. Statistical analysis was carried out using one-way ANOVA followed by Tukey’s post hoc test for multiple comparisons. In particular, the control was always compared with the formulations containing 25% additive, and additional comparisons were performed between the two additive concentrations (25% and 37.5%). In all comparisons: *p < 0.05, **p < 0.01, ***p < 0.001, and ****p < 0.0001.

## Supplementary Material



## Notes

Additional representative tensile curves for all blends, photos of samples characterized under compression, contact angle images, additional FTIR characterizations, representative temperature-dependent rheological curves for all samples, and supporting DSC curves (PDF)
