# Supplementary material for: Natural Blends of Ethyl Cellulose Oleogels for Tunable Bioplastic Design
Source: ACS Omega. 2026 Jan 27;11(5):8629–36. doi: 10.1021/acsomega.5c11908 (PMC12903159; doi:10.1021/acsomega.5c11908)
Supplement: Supplementary file 1 [file ao5c11908_si_001.pdf]

Exploration of Natural Blends in Ethyl Cellulose Oleogels for Tunable Bioplastic Design

Luca Cafuero[1], Marco Friuli[1], Muhammad Waheed[1], Christian Demitri[2], Alessandro Sannino[2], Carola Corcione[1], Leonardo Lamanna[1,2,*]

[1] Department of Engineering of Innovation

[2] Department of Experimental Medicine

*Corresponding author

Supporting Information

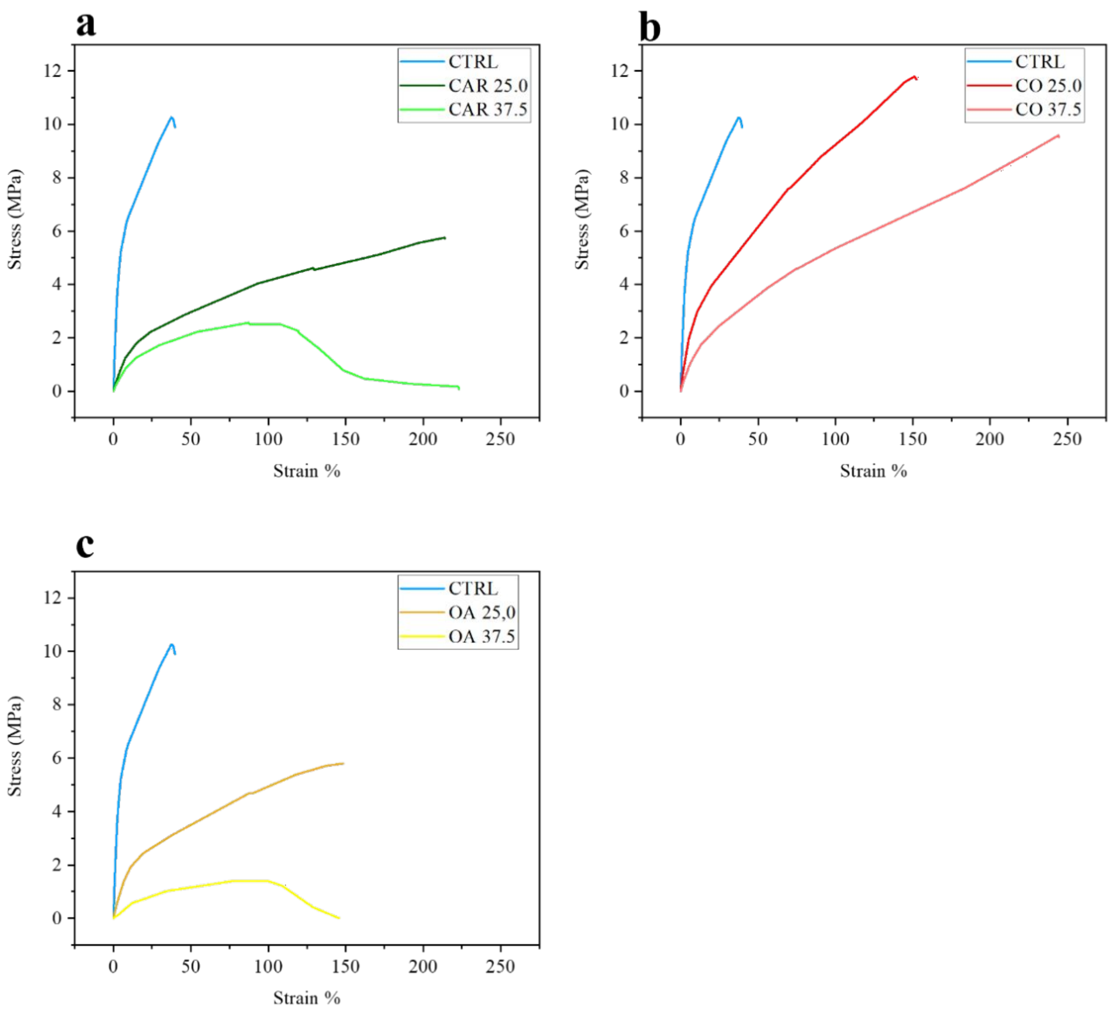

Figure S1: a-c) Representative curve for comparison between CTRL and new blends.

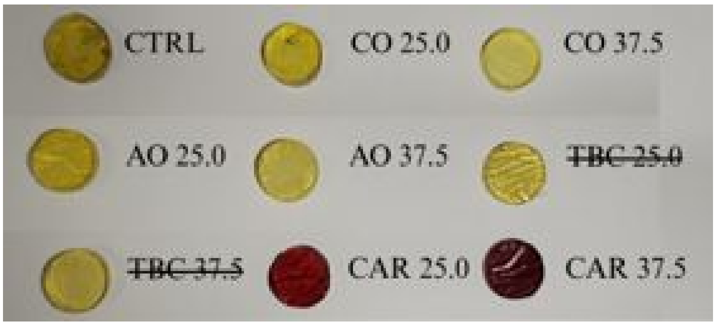

Figure S2: Representative samples for compression test.

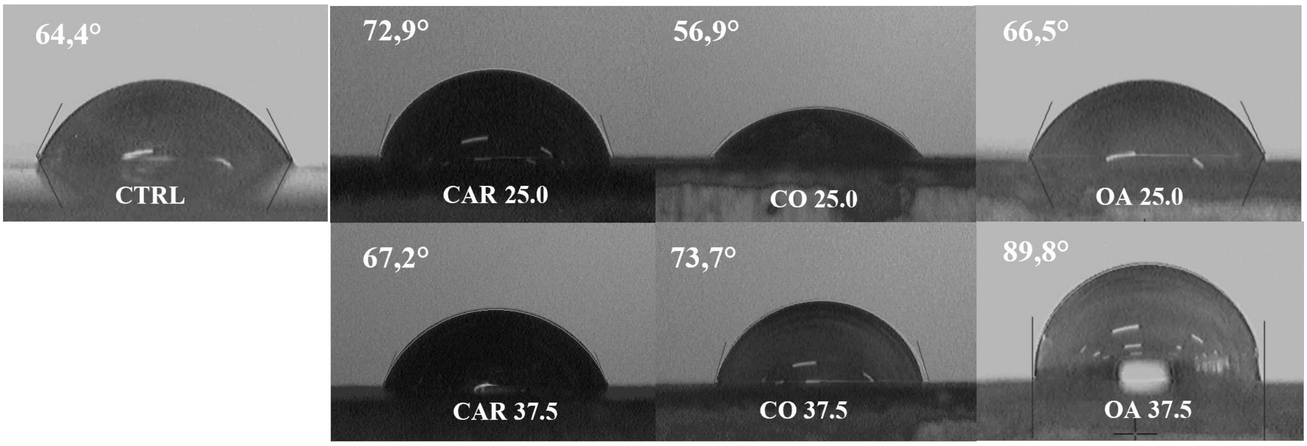

Figure S3: Representative sessile drops for CA measurements.

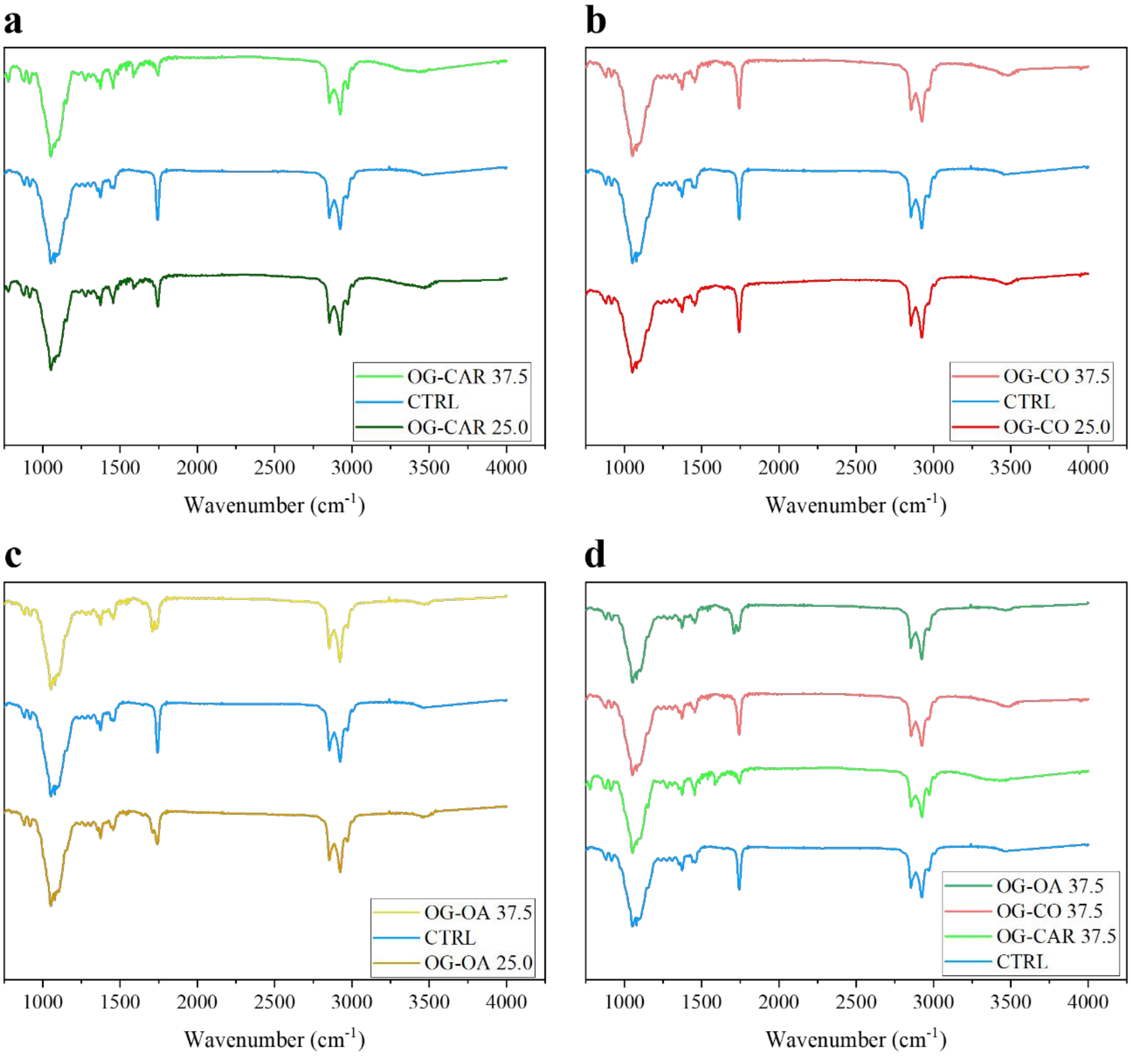

Figure S4: a- d) FTIR comparisons between CTRL and novelle formulations.

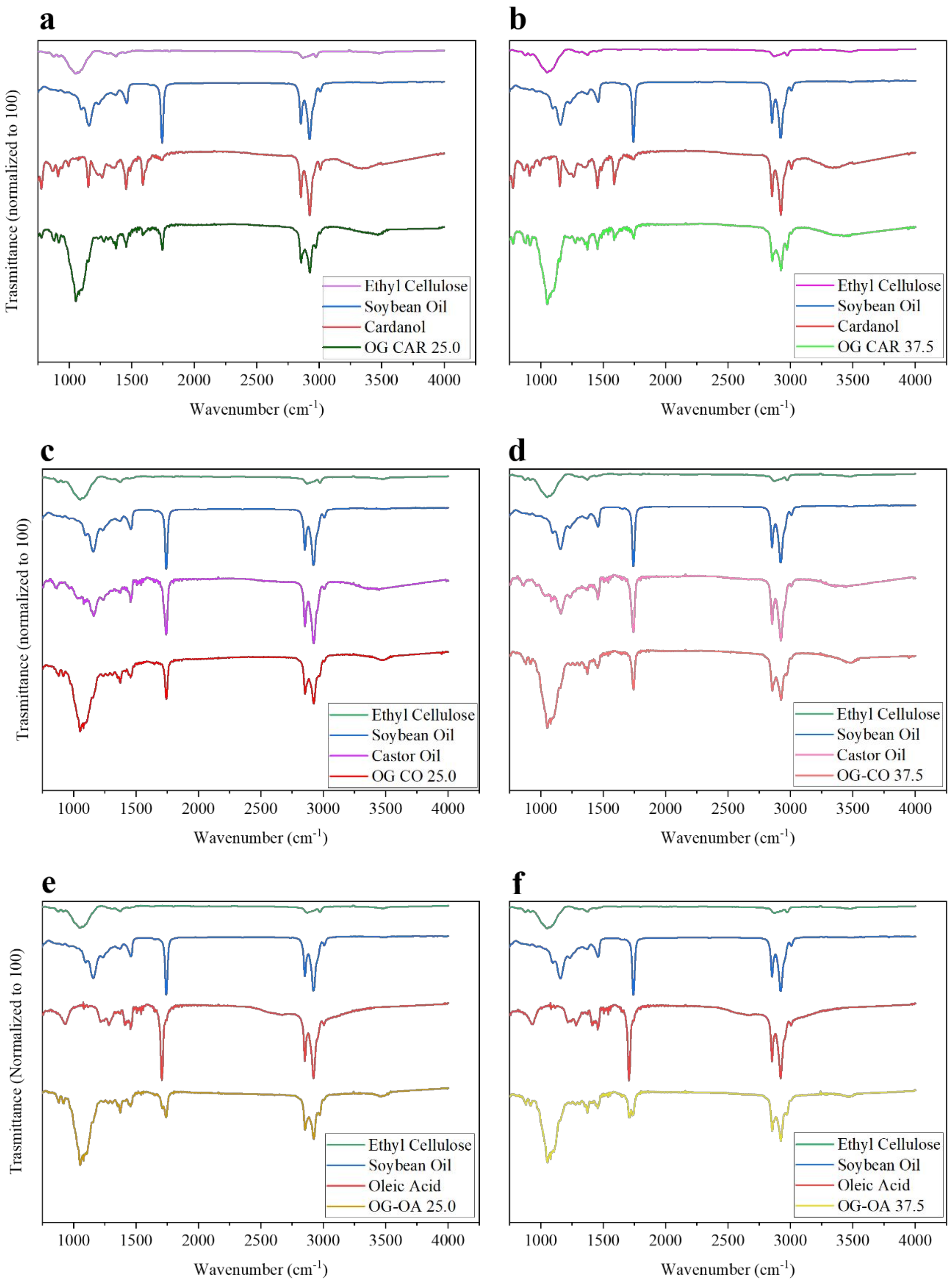

Figure S5: a)-f) FTIR of single component for each formulation.

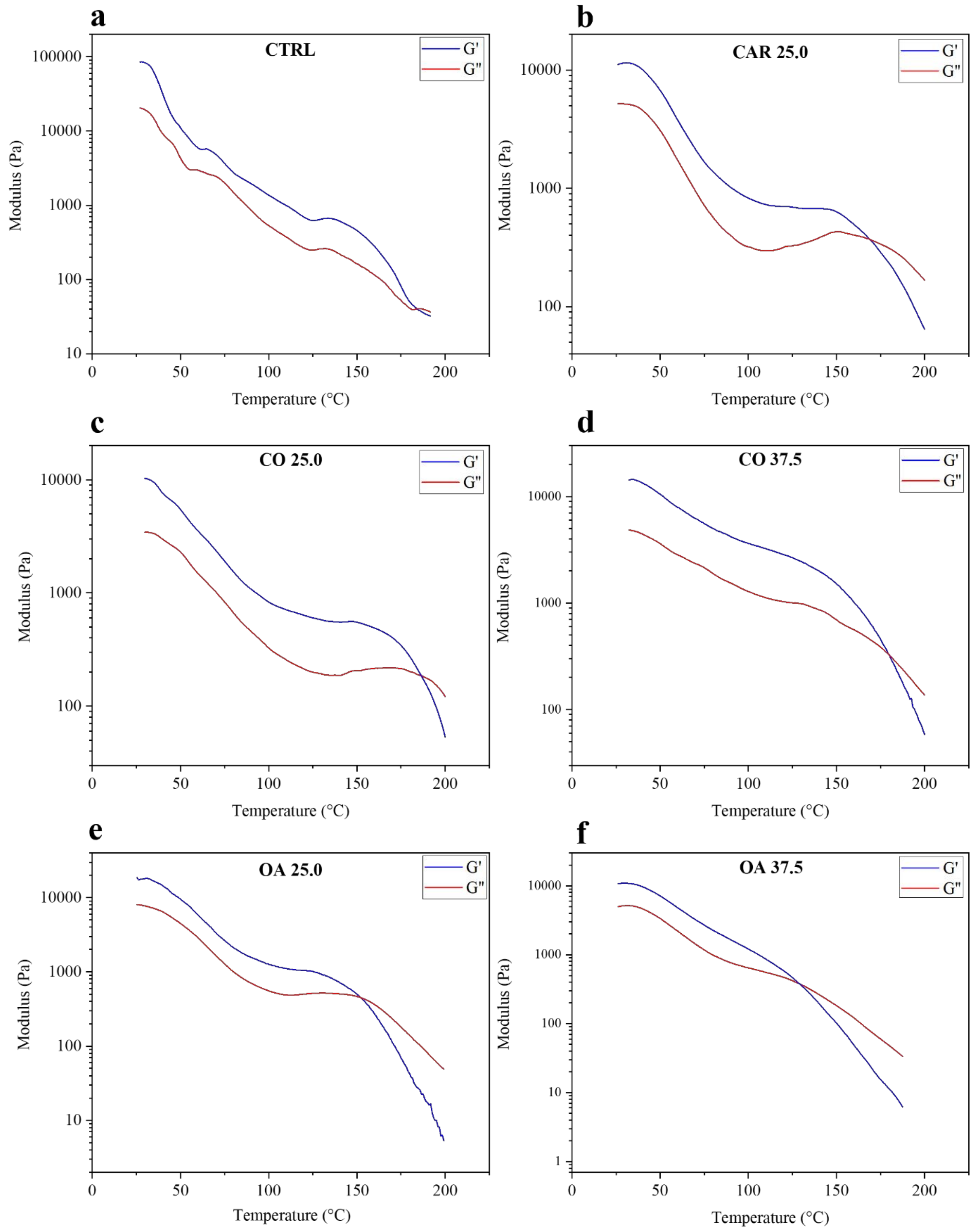

Figure S6: a- f) Representative curves for crossover temperatures for each formulation.

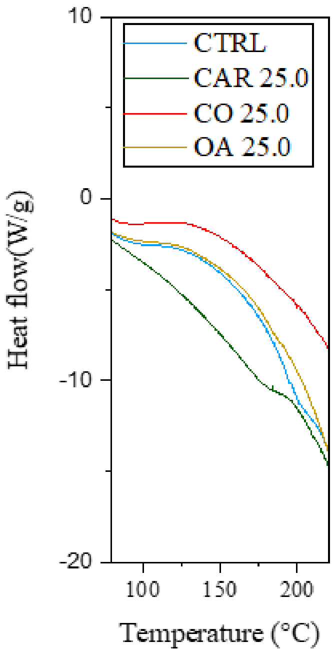

Figure S7: Second-order transitions are slightly visible as weak inflections in the DSC curves. In particular, they can be observed at ~195 °C for CTRL, ~180°C for CAR, ~200 °C for CO, and ~180 °C for OA, confirming the results obtained previously. The absence of pronounced peaks is attributed to the high oil content (50%), which smooths the thermal transitions of the polymer network.
